# Association of Anti-Smith, Anti-Ro, and Anti-ribonucleoprotein Combination With Accelerated Development of Lupus Nephritis in Systemic Lupus Erythematosus Patients in Saudi Arabia

**DOI:** 10.7759/cureus.75917

**Published:** 2024-12-17

**Authors:** Ahmed Alsalman, Taraq Albalawi, Faisael Albalwi, Majed Albirdisi

**Affiliations:** 1 Internal Medicine/Rheumatology, Dammam Medical Complex, Dammam, SAU; 2 Internal Medicine/Rheumatology, Prince Sultan Military Medical City, Riyadh, SAU; 3 Internal Medicine/Rheumatology, King Fahad Medical City, Riyadh, SAU

**Keywords:** anti-rnp antibodies, anti-ro/ssa, anti-smith antibodies, lupus nephritis, renal involvement in sle, systemic lupus erythromatosus

## Abstract

Introduction: Systemic lupus erythematosus (SLE) is an autoimmune inflammatory disorder affecting multiple organs. Lupus nephritis (LN), one of its serious complications, is characterized by proteinuria and renal dysfunction.

Objective: The objective of this study was to evaluate the association between a specific antibody profile (anti-Smith [anti-Sm], anti-Ro, and anti-ribonucleoprotein [anti-RNP]) and the time to develop LN in SLE patients.

Methods: A retrospective, single-center cohort study was conducted at King Fahad Medical City (KFMC) in Riyadh, Saudi Arabia, and included 128 patients with LN who visited the Rheumatology Clinic between 2014 and 2021. Patients were divided into two groups: a positive serological profile group, which included patients positive for all three antibodies (anti-Sm, anti-Ro, and anti-RNP), and a negative serological profile group (which included patients with at least one negative antibody result). Data on demographics, antibody profiles, time to proteinuria development, and LN classification were analyzed. The time to develop proteinuria from the initial diagnosis of LN to the first detection of proteinuria exceeding 500 mg was categorized into less than 1 year, 1-5 years, 6-10 years, and more than 10 years.

Results: Our findings revealed that a substantial proportion (95%) of patients positive for all three antibodies (anti-Sm, anti-Ro, and anti-RNP) had a significantly higher likelihood of developing proteinuria within the first five years of their SLE diagnosis, compared to 89.66% of patients with a negative serological profile.

Conclusion: The findings suggest that the presence of anti-Sm, anti-Ro, and anti-RNP antibodies is associated with a higher risk of early LN development, specifically within five years after initial SLE diagnosis. Regular monitoring and proactive management of high-risk patients can reduce the burden of LN and its complications.

## Introduction

Systemic lupus erythematosus (SLE) is a complex autoimmune inflammatory disease that impacts multiple organ systems and often results in severe, life-altering complications [1]. It predominantly affects young women and is driven by a combination of genetic predispositions and environmental triggers [2]. Renal involvement is one of the most serious manifestations, occurring in approximately 50% of individuals with SLE and frequently presenting as lupus nephritis (LN), which significantly impacts patient morbidity and mortality [3]. 

LN is characterized by immune-mediated glomerular injury resulting from the deposition of immune complexes, complement activation, and chronic inflammation [3]. The co-occurrence of certain autoantibodies may potentiate a more aggressive disease course, particularly in genetically predisposed populations, such as Africans [4]. Studies have found individuals of African descent to be disproportionately affected, showing both a higher prevalence of renal involvement and a tendency to develop nephritis earlier in the disease course [4]. Early detection and understanding of factors contributing to LN progression are critical for improving patient outcomes, yet the pathogenesis of LN remains incompletely understood.

Among the diverse immunological markers implicated in SLE, a specific triad of autoantibodies, anti-Smith (anti-Sm), anti-Ro, and anti-RNP, has been hypothesized to play a pivotal role in the onset and progression of LN [5]. The presence of these autoantibodies is strongly associated with immune dysregulation and heightened disease activity in SLE [5]. Anti-Sm antibodies, specific to SLE, are linked to severe disease phenotypes, while anti-Ro and anti-RNP antibodies are associated with systemic manifestations, including renal involvement [3].

Despite their clinical relevance, limited data exist on the combined effect of the anti-Sm, anti-Ro, and anti-RNP antibody triad in predicting LN progression. This study aims to address this gap by examining the prevalence of this antibody triad in Saudi patients with SLE and its correlation with the development and severity of LN. Through this work, we aim to provide a deeper understanding of the immunological factors driving LN progression and to inform targeted monitoring and management strategies for high-risk populations.

## Materials and methods

In this retrospective case-control study, 128 patients were recruited. The inclusion criteria comprised patients aged 14 years or older who visited the Rheumatology Clinic at King Fahad Medical City (KFMC) in Riyadh, Saudi Arabia, between 2014 and 2021. These patients met the American College of Rheumatology (ACR) and Systemic Lupus International Collaborating Clinics (SLICC) criteria for the diagnosis of SLE and LN. They had proteinuria greater than 0.5 g based on a 24-hour urine collection. The working diagnosis of LN with 500 mg/24 hours of proteinuria was not mandatory at the time of the first presentation. Instead, it was identified during the follow-up period as patients developed proteinuria above the threshold of 500 mg/24 hours. All patients meeting the inclusion criteria were included in the study, ensuring a representative cohort reflective of routine clinical practice. This process minimized selection bias and enhanced the transparency of the methodology. The exclusion criteria included patients younger than 14 years and those with other concomitant connective tissue diseases (MCTD). Patients with high titers of anti-RNP, along with the absence of features fulfilling the criteria for other systemic autoimmune diseases, were classified as MCTD and excluded from the study.

Patients were categorized into two groups: the first group consisted of patients with a positive serological profile (positive anti-Sm, anti-Ro, and anti-RNP antibodies), while the second group included patients negative for this antibody combination (had at least one negative antibody).

Demographic data, the class of LN based on renal histopathological specimens, and the duration of proteinuria development from the initial diagnosis of SLE to the first detection of proteinuria exceeding 500 mg were the variables analyzed. Twenty patients had suboptimal renal biopsy results that did not allow for reliable LN class identification and were therefore excluded from the study. This exclusion ensured that the analysis was based on accurate and robust histopathological classifications.

Descriptive statistics were employed to summarize patient characteristics and outcomes. Categorical variables were expressed as frequencies and percentages, while continuous variables were analyzed using independent t-tests. The statistical significance of categorical variables was determined using the chi-square test. Data analysis was performed using Statistical Analysis System (SAS) software, version 9.4 (SAS Institute Inc., Cary, NC), with a significance p-value set at ≤ 0.05. Survival analysis was conducted using the Kaplan-Meier method to estimate survival probabilities across groups categorized by gender (male and female) and nationality (Saudi and non-Saudi). This was done to evaluate whether these demographic factors impact survival probabilities or outcomes, since gender and ethnicity are among the most impactful predictors of LN outcomes [4]. Including these variables ensures that the findings are relevant to population-level disparities.

Ethical approval for the study was obtained from the King Fahad Medical City (KFMC) Research Committee, in accordance with the International Council for Harmonisation of Technical Requirements (ICH) Good Clinical Practice (GCP) guidelines.

## Results

The study included 124 Saudi patients and four non-Saudi patients diagnosed with LN, with the majority being female (85.94%) and males accounting for only 14.1% (Table 1). The median age of the participants was 30.5 years (Table 1).

**Table 1:** Demographics and clinical characteristics of the study population

	Frequency (%)
Ethnic group	
Saudi	124 (96.88%)
Non-Saudi	4 (3.13%)
Gender	
Female	110 (85.94%)
Male	18 (14.06%)
Kidney biopsy done	
Yes	127 (99.22%)
No	1 (0.78%)
ln class	
I	4 (3.70%)
II	11 (10.19%)
III	16 (14.81%)
III/V	9 (8.33%)
IV	43 (39.81%)
IV/V	10 (9.26%)
V	14 (12.96%)
VI	1 (0.93%)
Suboptimal biopsy	20
SM, RNP, and Ro status	
Positive (all three antibodies positive)	40 (31.25%)
Negative (at least one antibody negative)	88 (68.75%)
Duration from SLE diagnosis to proteinuria development	
Less than one year	32 (25.20%)
From one year to five years	84 (66.14%)
From 6 years to 10 years	9 (7.09%)
More than 10 years	2 (1.57%)
Age	Years
Mean	32.36
Median	30.50

The most common class of LN observed in this study was class IV (39.81%), followed by class III (14.81%) (Table 1). Forty patients (31.25%) tested positive for all three antibodies (anti-Sm, anti-Ro, and anti-RNP), while 88 patients (68.75%) tested negative for these antibodies (had at least one of these antibodies negative). Additionally, 20 patients had suboptimal renal biopsy results, which precluded LN class identification, and were therefore excluded from the study to ensure that the study's conclusions were based on reliable data from adequately assessed renal histopathology (Table 1).

Table 2 summarizes the frequency of each LN class in relation to the presence or absence of anti-Sm, anti-Ro, and anti-RNP antibodies. Among patients with positive antibody status, the most frequent LN classes were class IV (20.59%) and class V (20.59%), followed by class III (17.65%). In contrast, for patients with negative serological status, class IV was the most common (48.65%), followed by class III (13.51%). The p-value for the comparison of LN class distributions between positive and negative serological statuses was 0.0559, indicating a trend but not achieving statistical significance (Table 2).

**Table 2:** Frequency of lupus nephritis classes by anti-Sm, anti-Ro, and anti-RNP Status Anti-Sm: anti-Smith

LN class	SM, Ro, and RNP status		P-value
	Positive (%)	Negative (%)	0.0559
I	2 (5.88%)	2 (2.70%)	
II	5 (14.71%)	6 (8.11%)	
III	6 (17.65%)	10 (13.51%)	
III/V	5 (14.71%)	4 (5.41%)	
IV	7 (20.59%)	36 (48.65%)	
IV/V	2 (5.88%)	8 (10.81%)	
V	7 (20.59%)	7 (9.46%)	
VI	0 (0.00%)	1 (1.35%)	
Suboptimal biopsy = 20			

**Figure 1:**
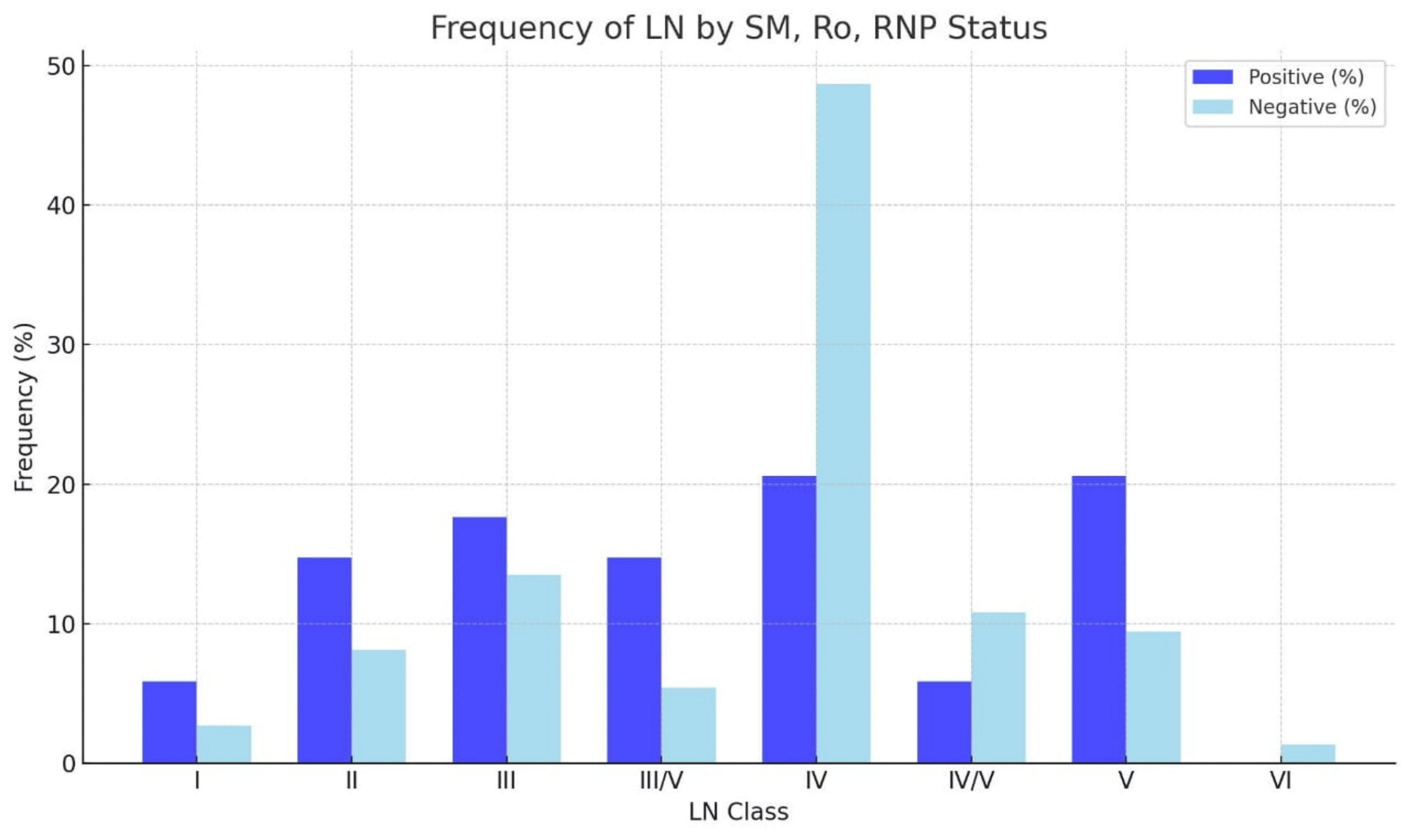
Frequency distribution of lupus nephritis classes by antibody status (SM, Ro, and RNP) SM: Smith

Figure 1 visually illustrates the frequency distribution of LN classes by anti-Sm, anti-Ro, and anti-RNP antibody status, divided into "Positive" and "Negative" categories. It emphasizes that Class IV was predominant in the "Negative" group, while Class V maintained a strong representation in the "Positive" group. Although the correlation between these variables was only marginally significant (p-value of 0.0559), these findings suggest that distinct antibody profiles may influence the progression or classification of LN.

Table 3 highlights the duration of proteinuria development, showing that patients with positive anti-Sm, anti-Ro, and anti-RNP antibodies developed proteinuria earlier compared to those without these antibodies. The p-value of 0.0033 indicates a statistically significant difference between the two groups regarding the onset of proteinuria.

**Table 3:** Proteinuria duration by antibody status (anti-Sm, anti-Ro, and anti-RNP) Anti-Sm: anti-Smith

Duration from SLE diagnosis to proteinuria development	SM, Ro, and RNP status		P-value
	Positive (%)	Negative (%)	0.0033
Less than one year	7 (17.50%)	25 (28.74%)	
From one year to five years	31 (77.50%)	53 (60.92%)	
From 6 years to 10 years	1 (2.50%)	8 (9.20%)	
More than 10 years	1 (2.50%)	1 (1.15%)	

There were seven patients (17.5%) with positive serological antibodies who developed nephritis within one year of SLE symptom onset, compared to 25 patients (28.74%) with negative serological titers (Table 3). In contrast, 31 patients (77.5%) with positive serological antibodies developed nephritis within one to five years of SLE diagnosis, compared to 53 patients (60.92%) with negative serological titers (Table 3).

The development of LN between 6 and 10 years after the initial SLE diagnosis was less common (Table 3). Specifically, one patient (2.5%) with positive serological titers and eight patients (9.2%) with negative serological titers developed LN during this period. Similarly, only one patient (2.5%) with positive serological titers and one patient (1.15%) with negative serological titers developed LN more than 10 years after the initial SLE diagnosis. These trends suggest that the majority of proteinuria cases occur within one to five years, regardless of antibody status, while longer durations (over six years) are rare (Table 3).

Table 4 summarizes survival time statistics for three groups: females, males, and the overall (combined) population. The mean survival time for females was 7.296 years, with a relatively wide confidence interval (4.793-9.798) and a median of five years. The mean survival time for males was slightly higher, at 7.4 years, with a narrower confidence interval (5.530-9.270) and a median of nine years. The median for males was significantly higher, indicating longer survival times compared to females. The mean survival time for the entire group was 7.281 years. In contrast, the median survival time matched that of females (5), suggesting that the female data had a greater weight in influencing the overall dataset (Table 4).

**Table 4:** Survival time estimates by gender

Gender	Mean estimate	Mean std. error	Mean 95% CI lower bound	Mean 95% CI upper bound	Median estimate	Median std. error	Median 95% CI lower bound	Median 95% CI upper bound
Female	7.296	1.277	4.793	9.798	5	0.924	3.189	6.811
Male	7.4	0.954	5.53	9.27	9	0		
Overall	7.281	1.194	4.941	9.622	5	1.992	1.097	8.903

**Figure 2:**
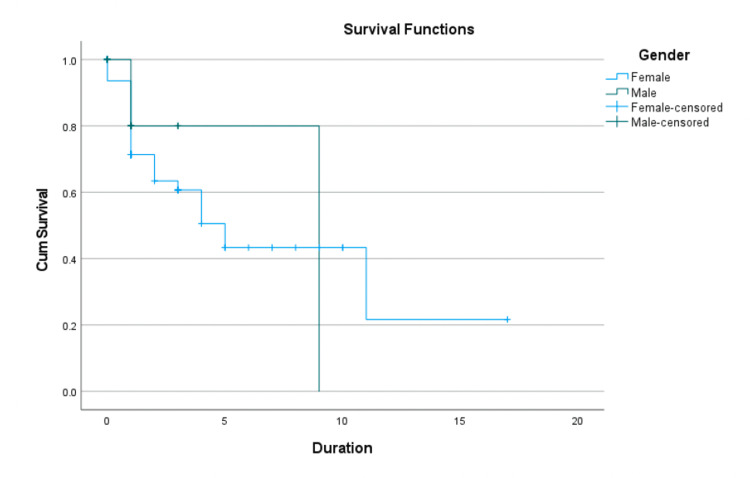
Kaplan-Meier survival curve by gender (female vs. male)

Figure 2 shows a Kaplan-Meier curve describing the probability of survival over time for males and females, including censored data (individuals who left the study or were still alive at the end of the observation period). The survival curves for males and females are relatively similar (Figure 2), as reflected in their comparable mean survival times (Table 4). A significant percentage of both groups (67% for females and 77.8% for males) were censored, indicating that their survival times were not fully complete. This limits the interpretability of median survival times, especially for males. The sharper decline in survival for females around the five-year time point aligns with their shorter median survival time (Figure 2). Overall, both males and females show comparable mean survival times; however, females experience earlier events (Figure 2).

Table 5 shows that the mean survival time was 7.467 years for Saudis (95% CI: 5.039-9.895), while the mean survival time for the non-Saudi group was four years, with a narrower confidence interval (1.600-6.4). Both groups had a median survival time of five years; however, for the Saudi group, the 95% confidence interval for the median was 0.639-9.361, indicating considerable uncertainty due to data variability or censoring. The mean survival time for the combined population was 7.281 years (95% CI: 4.941-9.622), closer to the Saudi group, suggesting that Saudi data may have a greater influence on the overall dataset. The median survival time for the combined population was also five years, with a confidence interval of 1.097-8.903, indicating moderate variability (Table 5).

**Table 5:** Survival time estimates by ethnicity

Ethnic group	Mean estimate	Mean std. error	Mean 95% CI lower bound	Mean 95% CI upper bound	Median estimate	Median std. error	Median 95% CI lower bound	Median 95% CI upper bound
Saudi	7.467	1.239	5.039	9.895	5	2.225	0.639	9.361
Non-Saudi	4	1.225	1.6	6.4	5	0		
Overall	7.281	1.194	4.941	9.622	5	1.992	1.097	8.903

The Saudi group consistently exhibited better survival probabilities compared to the non-Saudi group, as reflected in Table 5. This disparity is evident in the higher mean survival time for Saudis (7.467 years) compared to non-Saudis (4 years). This difference may stem from factors such as better access to healthcare, baseline health conditions, or demographic characteristics. The narrower confidence intervals for the non-Saudi group suggest less variability or fewer extreme survival times within that group. Overall, the survival statistics appear to be heavily influenced by the Saudi group, likely due to a larger sample size or greater weight in the dataset.

**Figure 3:**
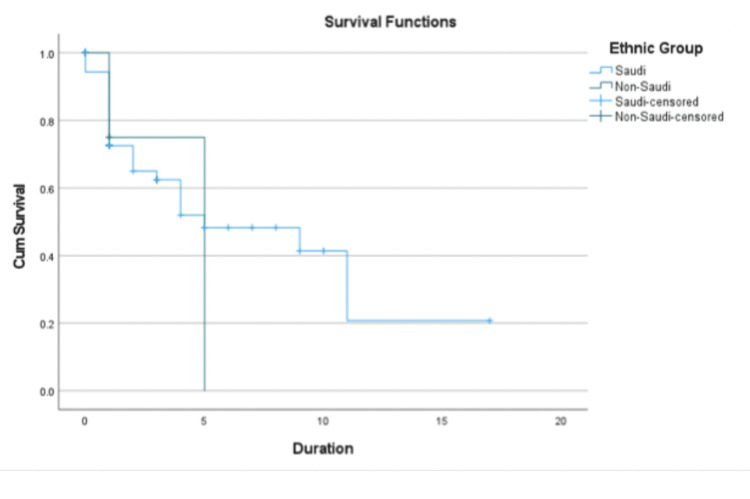
Kaplan-Meier survival curve by ethnic group (Saudi vs. non-Saudi)

Figure 3 presents a Kaplan-Meier survival curve comparing survival probabilities between Saudi and non-Saudi groups. The Saudi group demonstrates consistently higher survival probabilities compared to the non-Saudi group throughout the observation period.

Figure 3 further highlights the sharp drop in survival probabilities for the non-Saudi group in the earlier time periods, indicating a higher risk of earlier events compared to the Saudi group. However, these results should be interpreted cautiously due to the high proportion of censored data and wide confidence intervals. Overall, the survival trends and statistical estimates emphasize the need to consider underlying socio-demographic and clinical factors influencing survival outcomes.

## Discussion

Our retrospective study included 128 patients diagnosed with LN. The goal of our study was to determine the association between a positive anti-Sm, anti-Ro, and anti-RNP antibody triad and the time to develop LN following an initial SLE diagnosis. To the best of our knowledge, this is the first study to examine the duration of LN development post-SLE diagnosis in relation to the presence of the anti-Sm, anti-Ro, and anti-RNP triad within the Saudi Arabian population. The results showed that Saudi patients with a positive anti-Sm, anti-Ro, and anti-RNP antibody profile were at a higher risk of developing LN within five years of their initial SLE diagnosis.

In patients with active SLE, certain biomarkers can predict the likelihood of LN development. These include high levels of autoantibodies to nuclear antigens, such as anti-dsDNA, anti-Sm, anti-Ro, and anti-RNP, as well as hypocomplementemia, low levels of C1Q, and lymphopenia [6]. Potential pathways that these markers can accelerate LN progression include complement activation, immune complex deposition, and their downstream effects on glomerular inflammation and fibrosis. Our study found that 95% of patients with positive anti-Sm, anti-Ro, and anti-RNP triads developed LN within the first five years of SLE diagnosis (77.5% of which developed LN between one and five years), underscoring the prognostic value of these autoantibodies in identifying patients at risk for early renal involvement. Similarly, McCarty et al. reported that the serological triad of anti-Sm, anti-Ro, and anti-RNP was particularly prevalent in patients of African ancestry and was associated with severe and progressive renal disease [7]. Albirdisi et al. also found that patients with this autoantibody combination developed LN significantly earlier than those without it. Furthermore, they noted that this "autoantibody combination" was disproportionately represented in African ancestry patients [8]. Jordan and D’Cruz also concluded that the anti-Smith, anti-Ro, and anti-RNP combination was significantly more common in LN patients compared to non-nephritis patients [9]. They further emphasized that this antibody triad predisposed patients to an increased risk of advanced renal disease and shorter renal survival [9].

In contrast, Farid et al. reported that Bahraini patients with positive anti-Sm, anti-Ro, and anti-RNP antibodies but without anti-dsDNA had a lower risk of renal involvement [10]. Interestingly, the presence of anti-Sm/Ro/RNP antibodies appeared to be more common in the non-LN group, suggesting that these titers may have a protective role in certain populations [10]. Similarly, in 1998, the study by Al Attia et al. found no significant association between anti-Sm/Ro/RNP, anti-dsDNA, and LN development in Arab patients [11]. Ng also concluded that patients with this triple serology combination did not show a significant acceleration in the development of LN among Asian ancestry patients [12]. These conflicting findings may reflect differences in sample size, population characteristics (e.g., ethnicity, age distribution, baseline disease severity), and methodologies (e.g., study design, sample size, and statistical approaches).

Additionally, Alba et al. concluded that young Black patients with anti-dsDNA, anti-Sm antibodies, and positive lupus anticoagulant (LA) had a higher risk of renal involvement, suggesting that these patients warrant careful monitoring for LN development [13]. Kwon et al. further reported that high anti-dsDNA and anti-Sm antibody levels at the time of SLE diagnosis were associated with an increased future risk of LN development [6]. Migliorini et al. found that Anti-Sm antibodies were strongly correlated with the severity and activity of renal involvement [5]. In contrast, Hoffman et al. demonstrated that the presence of anti-RNP antibodies was associated with a lower risk of renal disease and appeared to have a protective effect against renal involvement in SLE [14].

Researchers have found that renal disease in SLE occurs "most often within five years of disease onset" and is one of the strongest predictors of poor outcomes [13]. Our study aligns with these findings, showing that LN development most commonly occurs within five years of an SLE diagnosis. Supporting this, Bastain et al. examined the timing of renal disease occurrence among different ethnic groups in America and found that two-thirds of Hispanic patients had evidence of renal disease at the time of SLE diagnosis [15]. Ng similarly showed that Malay patients had four higher odds of developing LN within the first five years after SLE onset compared to non-Malay patients [12].

These complementary and sometimes contrasting findings highlight the complexity of LN pathogenesis and the multifaceted role of autoantibodies in its progression. These results underscore the importance of considering additional factors, such as genetic predisposition, environmental influences, and treatment regimens, all of which may modulate disease progression.

Based on these implications, it is crucial to closely monitor SLE patients with anti-Sm, anti-Ro, and anti-RNP positivity for early signs of LN, particularly during the first five years following their SLE diagnosis. This antibody profile has been associated with a more aggressive disease phenotype, and therefore high-risk patients should undergo regular assessments for proteinuria and renal function to mitigate the risk of LN progression. Intensive follow-up during this critical period enables early detection and timely intervention, significantly reducing morbidity and mortality in SLE patients. Given the strong correlation between LN development and this initial timeframe, vigilant monitoring during the first five years is especially vital.

Limitations

While our study provides valuable insights into the relationship between anti-Sm, anti-Ro, and anti-RNP antibodies and the progression of LN in patients with SLE, several limitations must be acknowledged. The retrospective, single-center design and the small sample size for subgroups, combined with the exclusion of suboptimal biopsies, limit the study’s ability to establish a definitive causative relationship between the presence of specific autoantibodies and the accelerated progression of LN.

Although retrospective designs are prone to inherent biases (such as recall bias and incomplete records), our study provides a real-world snapshot of disease progression. It serves as a foundation for hypothesis generation. As the data were exclusively collected from KFMC in Saudi Arabia, the findings may lack generalizability to other populations, particularly those outside Saudi Arabia, due to potential genetic, environmental, or healthcare system differences. Certain subgroups, such as patients with less common LN classes or those with long-standing proteinuria before diagnosis, had relatively small participant numbers, which limited the statistical power of our analyses. Additionally, we considered the potential impact of suboptimal biopsy results and excluded such cases to ensure that our analysis was based on accurate and reliable histopathological classifications. Nevertheless, the majority of patients with complete biopsy data were representative of the studied population.

Other limitations include unaccounted confounders, ethnic homogeneity, and limited biomarker evaluation. Confounding factors influencing LN progression - such as medication adherence, comorbidities, and treatment regimens-were not analyzed in detail. However, the study’s focus on a well-defined cohort with specific autoantibody profiles minimizes variability and provides a clearer understanding of the relationship between these biomarkers and LN progression. Moreover, as the majority of participants were Saudi nationals, the findings may not be directly applicable to other populations. This limitation restricts the generalizability of the results to different ethnic or cultural groups without further validation in more diverse cohorts. However, the relatively homogeneous population studied has the advantage of reducing confounding variables, such as genetic or environmental differences. Similar studies conducted in diverse populations would help test the external validity of these findings. Furthermore, the study focused exclusively on anti-Sm, anti-Ro, and anti-RNP antibodies without accounting for other potentially relevant biomarkers or complement levels, which could provide additional insights into LN progression.

Future studies should address these limitations by employing multicenter, longitudinal designs with larger, more diverse patient populations and comprehensive biomarker analyses. Such research would enhance our understanding of the relationship between autoantibodies and LN. While our study is retrospective and single-centered, it provides a focused and practical exploration of LN progression within the Saudi population. By analyzing a large cohort with robust data on serological markers, our findings contribute clinically relevant insights aligned with global trends reported in similar studies.

## Conclusions

This study underscores the association between anti-Sm, anti-Ro, and anti-RNP antibodies and the accelerated development of LN in patients with SLE. Notably, our study concluded that a substantial portion of patients with these antibodies developed proteinuria within five years of SLE diagnosis. These autoantibodies may serve as valuable markers for identifying patients at risk of early LN development, highlighting the importance of proactive monitoring and management in these individuals.

Future research should build on these insights through multi-center, longitudinal studies involving diverse populations to validate and refine these observations. Additionally, investigations into additional biomarkers would provide a more comprehensive understanding of LN pathogenesis, enabling more effective and personalized approaches to treatment.

## References

[1] Carter EE, Barr SG, Clarke AE (2016). The global burden of SLE: prevalence, health disparities and socioeconomic impact. Nat Rev Rheumatol.

[2] Leng X, Xia J, Zeng X, Song Y (2020). Prevalence and associated factors of lupus in the United States: Third National Health and Nutritional Examination Survey (NHANES III). Front Med (Lausanne).

[3] Almaani S, Meara A, Rovin BH (2017). Update on lupus nephritis. Clin J Am Soc Nephrol.

[4] Lea JP (2002). Lupus nephritis in African Americans. Am J Med Sci.

[5] Migliorini P, Baldini C, Rocchi V, Bombardieri S (2005). Anti-Sm and anti-RNP antibodies. Autoimmunity.

[6] Kwon OC, Lee JS, Ghang B, Kim YG, Lee CK, Yoo B, Hong S (2018). Predicting eventual development of lupus nephritis at the time of diagnosis of systemic lupus erythematosus. Semin Arthritis Rheum.

[7] McCarty GA, Harley JB, Reichlin M (1993). A distinctive autoantibody profile in black female patients with lupus nephritis. Arthritis Rheum.

[8] Albirdisi M, D’cruz D, Sangle S, Jordan N (2020). Autoantibody profile and ethnicity: risk factors for accelerated development of lupus nephritis. Ann Rheum Dis.

[9] Jordan N, D’Cruz D (2013). Predictive value of the autoantibody triad of anti-Ro, anti-Sm and anti-RNP for the future development of lupus nephritis. Am Coll Rheumatol.

[10] Farid EM, Hassan AB, Abalkhail AA, El-Agroudy AE, Arrayed SA, Al-Ghareeb SM (2013). Immunological aspects of biopsy-proven lupus nephritis in Bahraini patients with systemic lupus erythematosus. Saudi J Kidney Dis Transpl.

[11] Al Attia HM, Al Ahmed YH, Chandani AU (1998). Serological markers in Arabs with lupus nephritis. Lupus.

[12] Ng CR (2022). Role of combination autoantibodies (anti-Smith, Ro and RNP antibodies) and ethnicity in accelerated development of lupus nephritis. Ann Rheum Dis.

[13] Alba P, Bento L, Cuadrado MJ (2003). Anti-dsDNA, anti-Sm antibodies, and the lupus anticoagulant: significant factors associated with lupus nephritis. Ann Rheum Dis.

[14] Hoffman I, Peene I, Meheus L (2003). Presence of anti-RNP-A and anti-RNP-C antibodies is inversely associated with renal symptoms of systemic lupus erythematosus. Arthritis Res Ther.

[15] Bastian HM, Roseman JM, McGwin G Jr (2002). Systemic lupus erythematosus in three ethnic groups. XII. Risk factors for lupus nephritis after diagnosis. Lupus.

